# Growth phase is an important, species-specific determinant of yeast adhesion to abiotic surfaces

**DOI:** 10.1038/s41598-026-48411-8

**Published:** 2026-05-14

**Authors:** Vítězslav Plocek, Jana Maršíková, Vichi Sicha Irianto, Libuše Váchová, Zdena Palková

**Affiliations:** 1 Faculty of Science, Charles University, BIOCEV, Prague, Czech Republic; 2 Institute of Microbiology of the Czech Academy of Sciences, BIOCEV, Prague, Czech Republic

**Keywords:** Yeast adhesion, *Candida* spp., *Saccharomyces cerevisiae*, Exponential- and stationary-phase cells, Cell morphology, Biological techniques, Biotechnology, Microbiology

## Abstract

Different yeast species differ markedly in their ability to adhere to solid abiotic surfaces and form surface-associated biofilms. Yeast adhesion is influenced by multiple factors, including the genetically determined expression of specific adhesion proteins, the physiological state of the cells that determines whether adhesive properties are expressed, and environmental conditions such as growth medium and surface characteristics. In this study, we investigated how adhesion to a plastic (polystyrene) surface is affected by growth phase (exponential versus stationary) and cell morphology (yeast-form cells versus hyphae/pseudohyphae) in several *Candida* species and in selected clinical and wild strains of *Saccharomyces cerevisiae*, under different growth media conditions. We show that for most *Candida* species and *S. cerevisiae*, exponential-phase cells adhered to polystyrene more efficiently than stationary-phase cells. In contrast, both *C. glabrata* strains displayed the opposite trend – higher adhesion efficiency of stationary-phase cells than exponential-phase cells. A clear association between cell morphology and adhesion was observed only in *C. albicans* where hyphae or pseudohyphae adhered efficiently to polystyrene, whereas yeast-form cells were poorly adhesive. This morphology-dependent adhesion was not detected in other *Candida* species or in *S. cerevisiae*. Our results demonstrate that growth phase is an important, species-specific determinant of yeast adhesion to abiotic surfaces.

## Introduction

Yeast cell adhesion and biofilm formation on abiotic surfaces such as stainless steel, plastic, and glass is a critical phenomenon in both industrial and clinical settings. This complex, multi-step process involves yeast cells adhering to surfaces, proliferating, and producing extracellular polymeric substances, resulting in robust structures that are highly resistant to removal. Biofilm formation by pathogenic yeasts, such as various *Candida* spp., on medical devices like catheters and prostheses can cause infections with high mortality rates. Yeast biofilms that develop on equipment, pipelines, and packaging lead to contamination, spoilage, and significant economic losses, especially in the dairy, brewing, and beverage industries^1,2^.

The formation of microbial biofilms on abiotic surfaces (e.g., plastic, glass, or metal) begins with the adhesion of cells to a solid surface, followed by the development of a complex three-dimensional biofilm structure at the solid-liquid interface^3–7^. The ability of microbial cells to attach to a solid surface depends on multiple factors, including intrinsic cellular properties, the physicochemical characteristics of the biotic or abiotic surface – such as surface free energy, chemical composition, roughness, hydrophobicity, and surface charge – and environmental factors, such as temperature, pH, and the composition of the surrounding medium^8^.

Yeast cell adhesion has been studied primarily in major yeast pathogens, such as *Candida albicans*, as well as in the model organism *Saccharomyces cerevisiae*^5,9–14^. Yeast adhesion is mediated in part by specific cell surface proteins known as adhesins (e.g., ALS proteins in *C. albicans* and Flo11 in adhesive strains of *S. cerevisiae*)^13–18^. These adhesins are encoded by specific genes – their presence on the cell surface therefore depends on whether their genes are expressed under given environmental conditions. In addition to genetically determined adhesins, yeast adhesion is also influenced by more complex and less gene-specific properties of the cell surface, particularly features of the yeast cell wall. These include its hydrophobic or hydrophilic character, the presence of cell wall polysaccharides with different adhesive properties, and other surface-related traits^1,18,19^. Adhesion is further affected by general cellular characteristics, including cell size, which can vary across different stages of the cell cycle as well as during transitions between distinct developmental programs, such as the switch between yeast-like and hyphal or pseudohyphal growth (the so-called dimorphic transition)^20^. All of these parameters can influence the ability of cells to adhere to a wide range of biotic and abiotic surfaces, whose physical and chemical properties likewise determine whether a given cell type can attach^1^.

Numerous studies have also demonstrated that the physiological state of adhering cells, determined by growth conditions in a particular medium (such as nutrient availability), represents an important factor influencing cell adhesion^1^. There is less information about the influence of the specific growth phase. These conditions can substantially modify cell surface properties. The current state of knowledge on factors affecting microbial adhesion has been summarized in several recent review articles; however, these reviews also highlight considerable fragmentation of available data^1^. This fragmentation arises from the use of different growth media, diverse biotic and abiotic surfaces, and distinct yeast species or strains across studies, as well as from the fact that most research has focused on only a limited number of yeast species and a single growth phase.

In this study, we addressed straightforward questions: how is yeast adhesion to an abiotic polystyrene surface influenced by the growth phase (exponential and stationary), and how does this effect differ among yeast species and under different growth media conditions? A further question concerned the role of dimorphic switching and specific cellular morphotypes (yeast-form cells versus hyphae or pseudohyphae) in adhesion efficiency. For this analysis, we selected three adhesive strains of *S. cerevisiae*, a non-adhesive laboratory strain of *S. cerevisiae* and five different *Candida* species, grown in four different media and harvested at different growth phases. The media were chosen based on previous experiments showing that yeast cells precultivated in these media differ in their adhesive properties and biofilm-forming capacity^11^.

Comparison of cells in the logarithmic growth phase (exponential cells) and stationary-phase cells revealed differences in adhesion efficiency that were characteristic of individual yeast species and, in some cases, further modulated by cultivation in specific media. In contrast, the observed differences in adhesion between exponential- and stationary-phase cells were not related to the specific morphological form of the cells (yeast-form cells versus hyphae or pseudohyphae).

## Results

### Growth rates and biomass accrual in different media in selected yeast genera and strains

We selected four *S. cerevisiae* strains and six strains of *Candida* species (Table 1). The *S. cerevisiae* wild strain BR-F and two clinical strains, YJM320 and YJM789, are able to adhere to plastic surfaces and form structured biofilms, whereas the laboratory strain BY4742 is a non-adhesive control strain^11,21^. The *Candida* set included two strains of *C. glabrata* (CBS138, isolated from feces, and DSM11226, isolated from blood), *C. albicans* (DSM11225, isolated from human blood), *C. krusei* (DSM6128, isolated from a sputum sample from a patient with bronchomycosis), *C. parapsilosis* (DSM5784, isolated from a human case of sprue), and *C. tropicalis* (DSM5991).

**Table 1:** Strains used in this study.

Yeast	Strain	Source	Collection No.
*S. cerevisiae*	BY4742	Euroscarf	Y10000
*S. cerevisiae*	BR-F	Collection of the Institute of Chemistry, Slovak Academy of Sciences	CCY 21-4-97
*S. cerevisiae*	YJM320	Phaff Yeast Culture Collection	11–133
*S. cerevisiae*	YJM789	Phaff Yeast Culture Collection	15–436
*C. glabrata*	CBS138	ATCC collection	2001
*C. glabrata*	DSM11226	DSMZ collection	11,226
*C. albicans*	DSM11225	DSMZ collection	11,225
*C. krusei*	DSM6128	DSMZ collection	6128
*C. tropicalis*	DSM5991	DSMZ collection	5991
*C. parapsilosis*	DSM5784	DSMZ collection	5784

**Fig. 1:**
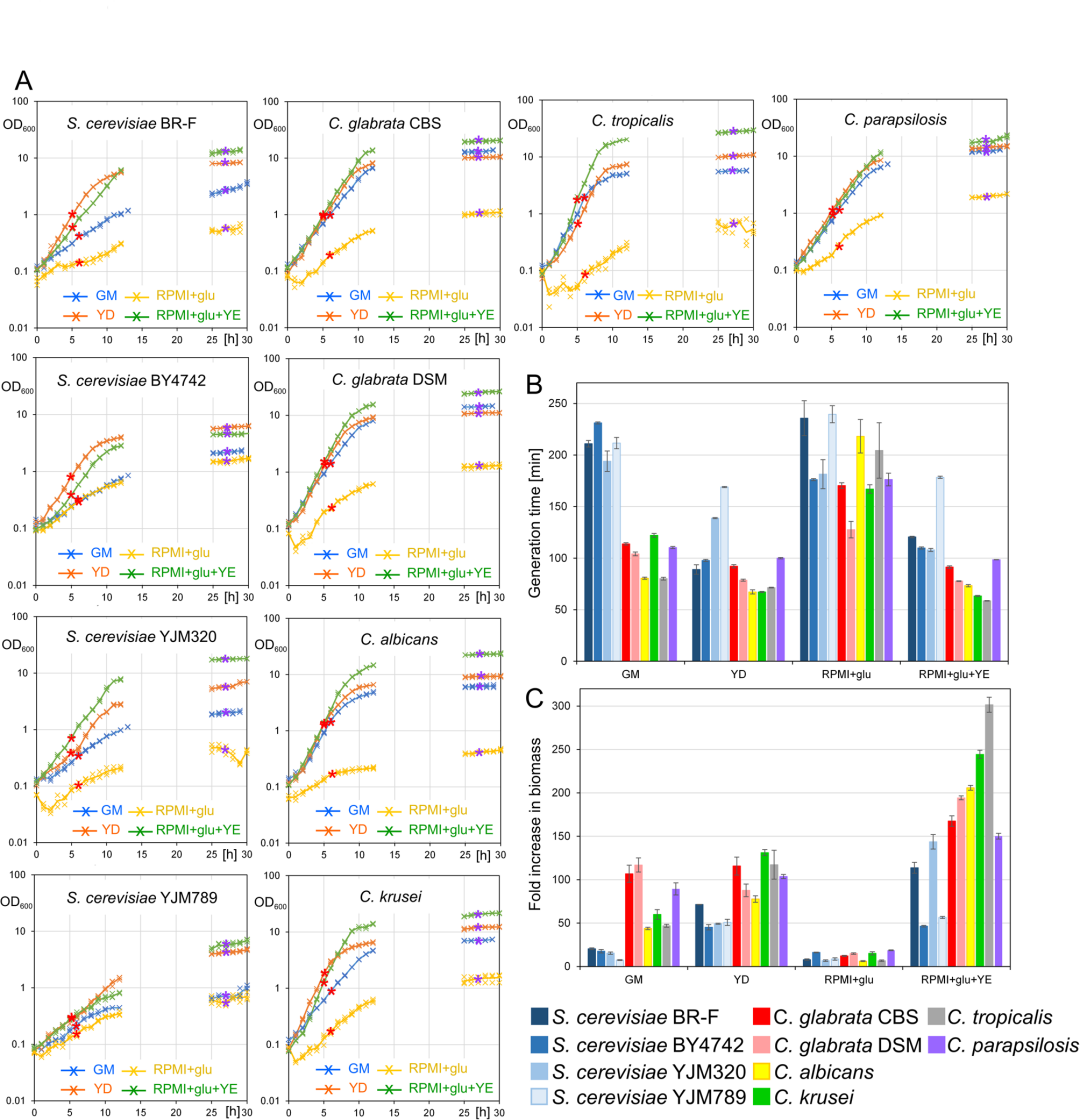
Growth characteristics of *S. cerevisiae* and *Candida* spp. strains in different media. (**A**) Growth curves of individual strains cultivated in four media. OD_600_ values at individual time points from three independent biological replicates are shown; curves represent mean values. Stars indicate time points at which cells were harvested for morphology and adhesion analyses (red star, exponential phase; violet star, stationary phase; see Figs. 2 and 4). (**B**) Mean generation times calculated from the exponential phase of growth for each strain and medium. (**C**) Mean fold biomass increase in culture OD_600_ between inoculation (t_0_) and stationary phase (t_25_) calculated as OD_600_ (t_25_) divided by OD_600_ (t_0_). Data in panels (**B**) and (**C**) represent means of three independent biological replicates with standard deviations (SD) and are based on analysis of the growth curves in (**A**).

We first focused on defining growth conditions for the analysis of exponential- and stationary-phase cells (Fig. 1). We examined the growth characteristics and cell morphologies of *S. cerevisiae* and *Candida* strains in four different liquid media which differ in complexity and carbon source. The respiratory medium GM is a complex medium containing glycerol and yeast extract (YE), while the glucose medium YD is a complex medium containing 2% glucose and YE; these two media differ only in the carbon source. The third medium, RPMI 1640 supplemented with 2% glucose (RPMI+glu), is a synthetic defined medium commonly used for analyses of adhesion and biofilm formation in *Candida* species. The fourth medium, RPMI+glu+YE, consists of RPMI+glu supplemented with 1% YE, corresponding to the YE concentration in YD. Thus, RPMI+glu+YE differs from YD only by the presence of RPMI components.

Both yeast growth rates (generation time during exponential growth) and the fold increase in biomass (OD_25h_/OD_0h_) over a defined cultivation interval (0–25 h) differed markedly among the tested media (Fig. 1). All strains grew slowly in the synthetic RPMI + glu medium (Fig. 1B), which was also associated with a low biomass fold increase (Fig. 1C). The shortest and comparable generation times for all *Candida* species were observed in the glucose- and YE-containing media (YD and RPMI+glu+YE). However, the biomass fold increase differed substantially between these two media and was markedly higher in RPMI+glu+YE than in YD (Fig. 1B, C). Compared to YD, growth in the respiratory GM medium resulted in a 10–30% longer generation time (i.e., lower growth rate) for all *Candida* species except *C. krusei*, for which the prolongation was much more pronounced (approximately 180%). In GM, the biomass fold increase reached 40–60% of that observed in YD for *C. albicans*, *C. krusei*, and *C. tropicalis*. In contrast, for *C. parapsilosis* and *C. glabrata* CBS, the increase in GM was nearly identical to that in YD (approximately 90%), and for *C. glabrata* DSM it exceeded the YD value (approximately 130%). Among all tested strains, the two *C. glabrata* strains exhibited the highest fold increase in biomass in GM medium.

In contrast to *Candida* species, the growth rates and biomass fold increases of *S. cerevisiae* strains varied more strongly across media and, in some cases, also between strains grown in the same medium. All four *S. cerevisiae* strains displayed markedly longer generation times and a lower overall fold increase in biomass in GM compared to YD. For strains BR-F and BY4742, the generation time in GM was approximately 235% of that in YD, whereas the difference was smaller for the two clinical isolates, reaching 125–140% of the YD generation time. In GM, the fold increase ranged from 15% to 40% of the YD value across all four strains. In YD medium, generation times were comparable to those in RPMI+glu+YE for BY4742 and YJM789, shorter for BR-F (approximately 70%), and longer for YJM320 (approximately 130%). The biomass fold increase in YD and RPMI+glu+YE was similar for BY4742 and YJM789 but was substantially higher in RPMI+glu+YE compared to YD for BR-F (approximately 160%) and particularly for YJM320 (approximately 290%).

### *Candida albicans* exhibits a marked morphological response to changes in growth conditions

Based on the growth profiles (growth curves) of individual strains (Fig. 1A), we defined cultivation times corresponding to the mid-exponential growth phase (after 5–6 h of cultivation, indicated by a red asterisk in Fig. 1A) and the stationary phase (after 27 h of cultivation, indicated by a purple asterisk in Fig. 1A).

**Fig. 2:**
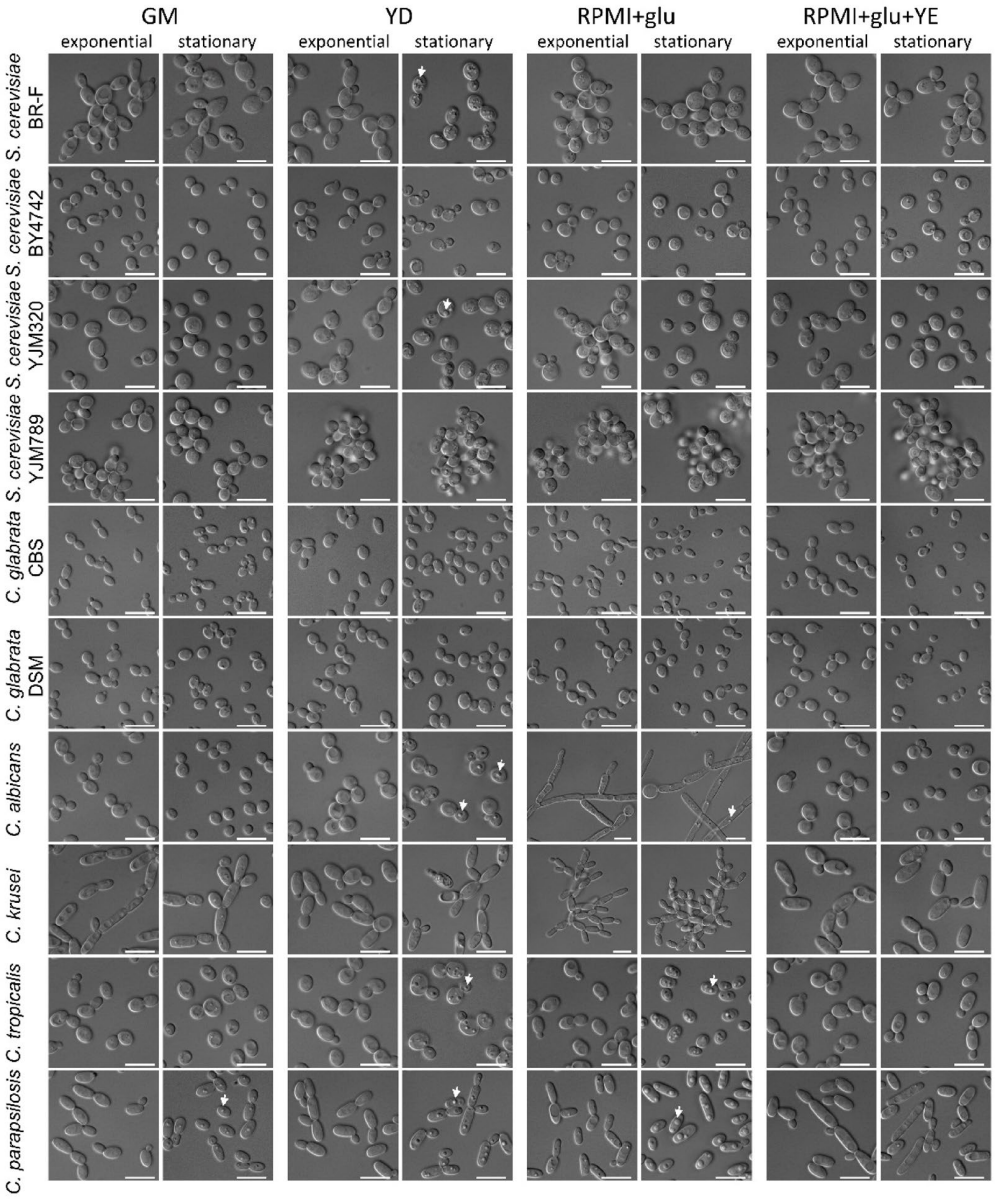
Morphology of exponential- and stationary-phase cells of *S. cerevisiae* and *Candida* spp. Cells harvested in the exponential phase (red asterisk in Fig. 1) and stationary phase (violet asterisk in Fig. 1) were visualized by differential interference contrast (DIC) microscopy. White arrows indicate intracellular bodies of variable size observed in some stationary-phase cells, corresponding to lipid droplets (see Fig. 3). Scale bar, 10 μm.

Cells of all tested yeast strains in these two distinct growth phases – mid-exponential and stationary (Fig. 1A) – were harvested from each medium and their morphology was examined by light microscopy using differential interference contrast (DIC) imaging (Fig. 2). Microscopic analysis revealed clear morphological differences among individual strains. In contrast, the effects of growth medium and growth phase on the cell morphology of individual strains were generally modest (Fig. 2). The most pronounced – and, in fact, the only clearly medium-dependent – morphological change was observed in *C. albicans*. In GM, YD, and RPMI+glu+YE, *C. albicans* formed almost exclusively oval to round yeast-form cells. In striking contrast, growth in the synthetic RPMI+glu medium resulted in the formation of very long, highly branched true hyphae, with occasional long pseudohyphae. This difference was maintained in both exponential and stationary *C. albicans* cells.

Yeast-form cells clearly predominated across all media and in both growth phases in *S. cerevisiae* strains BY4742 and YJM320, both *C. glabrata* strains, and *C. tropicalis* (Fig. 2). The clinical *S. cerevisiae* strain YJM789 formed prominent clusters of oval yeast-form cells, from which individual cells were released; these clusters were observed in all media and in both growth phases. Cells of the *S. cerevisiae* strain BR-F were predominantly oval but frequently remained connected and formed pseudohyphae of variable length and branching. In addition to pseudohyphae, individual cells, short chains of cells, and multicellular aggregates were also observed. In *C. krusei*, branched pseudohyphae of varying length predominated in all tested media, often in combination with elongated yeast-form cells. Larger clusters of branched pseudohyphae were particularly evident in RPMI+glu, whereas relatively fewer pseudohyphae were observed in RPMI+glu+YE and YD. Cells of *C. parapsilosis* were elongated in all media, with occasional formation of short, sometimes branched pseudohyphae; no consistent medium-dependent differences in morphology were detected.

**Fig. 3:**
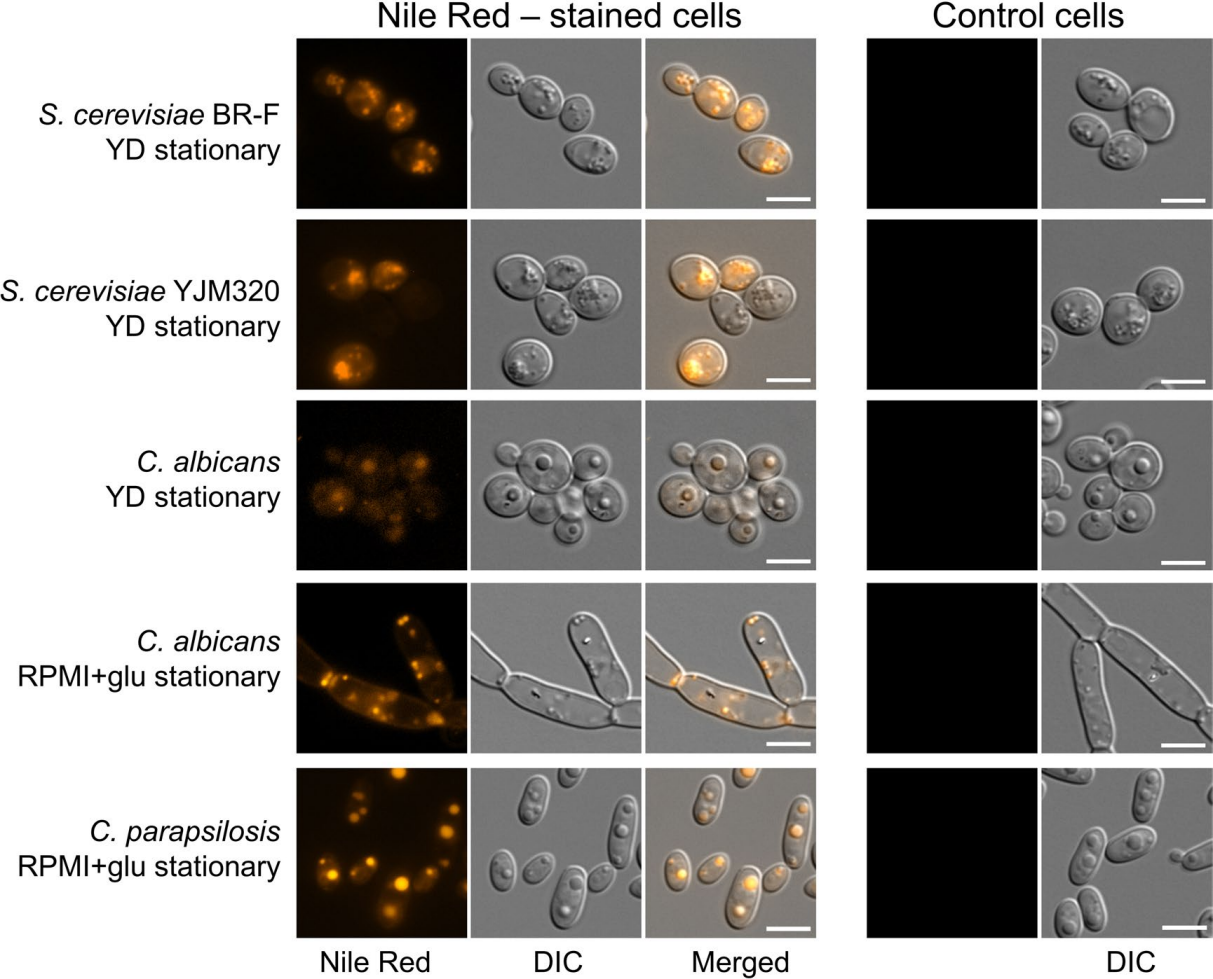
Lipid droplets in stationary-phase cells. Stationary-phase cells of selected strains were stained with Nile Red dye and observed using fluorescence microscopy combined with DIC (left panel). Control unstained cells are in the right panel, which shows the absence of autofluorescence (left image, taken under the same conditions as for Nile Red imaging) and DIC visualization (right image). Scale bar, 10 μm.

No significant differences in cell morphology (yeast-form cells versus hyphae or pseudohyphae) were observed between exponential- and stationary-phase cells in any of the tested media (Fig. 2). However, stationary-phase cells of several strains contained prominent intracellular structures that were absent or rare in exponential-phase cells. These structures varied in size and were observed, for example, in *C. albicans* grown in YD or RPMI+glu, *C. tropicalis* grown in YD and RPMI+glu, *C. parapsilosis* grown in GM, YD, and RPMI+glu and *S. cerevisiae* BR-F and YJM320 grown in YD (Fig. 2, white arrows). Staining of cells of selected strains with the lipid-specific dye Nile Red revealed that these intracellular structures (both small and large) are most likely lipid droplets of variable size formed during stationary phase (Fig. 3). Lipid droplets are intracellular storage structures for non-polar lipids, primarily triacylglycerols and steryl esters, which begin to form during early stationary-phase^22^.

**Fig. 4:**
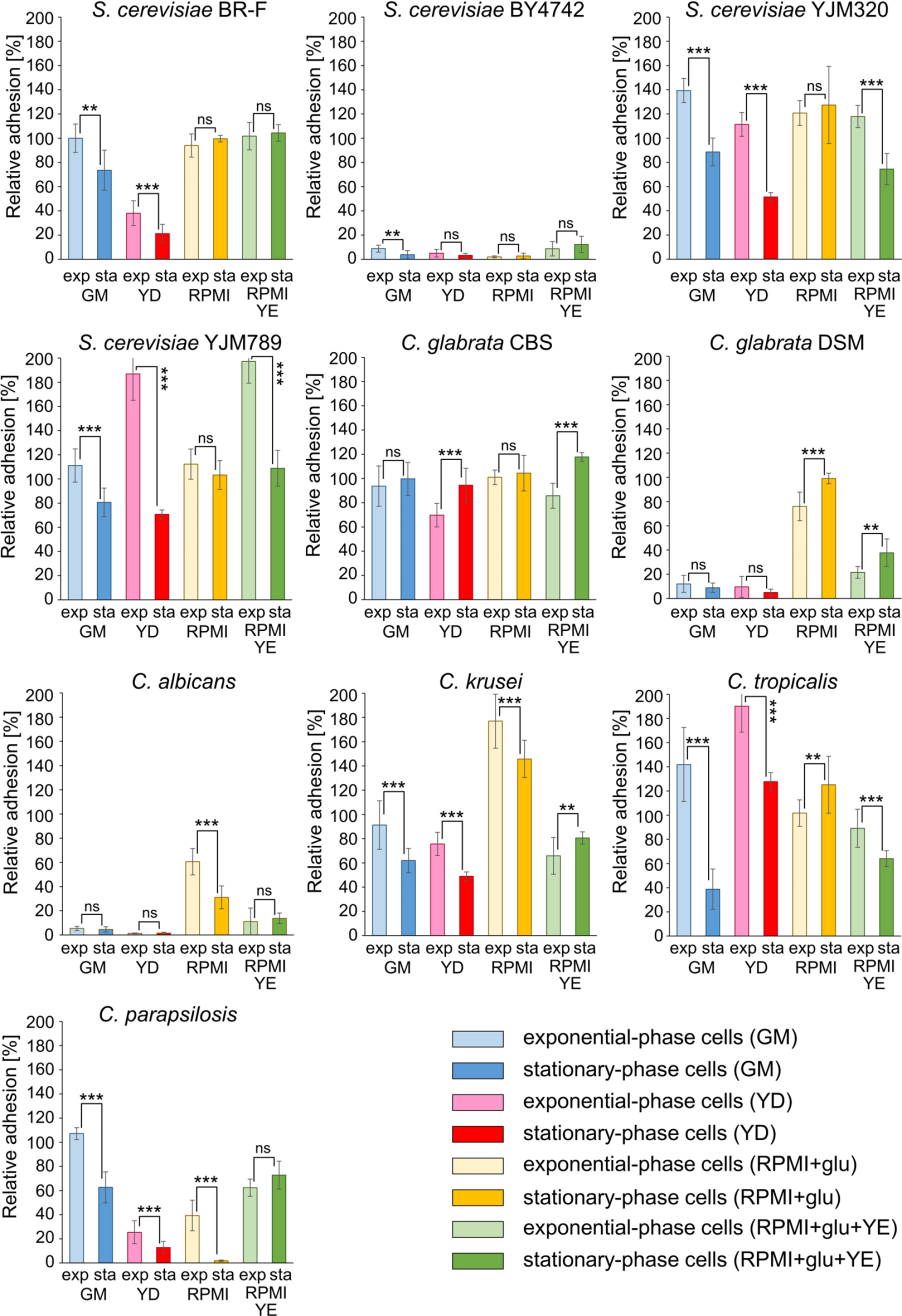
Relative adhesion of exponential- and stationary-phase cells to a polystyrene surface. Exponential-phase cells (exp) (red asterisk in Fig. 1) and stationary-phase cells (sta) (violet asterisk in Fig. 1) were used for adhesion assays. Data represent mean values from at least eight independent replicates with standard deviations (SD). Statistical significance was assessed using a two-tailed unpaired t-test: ***, *p* < 0.001; **, *p* < 0.01; *, *p* < 0.05; ns, not significant.

**Fig. 5:**
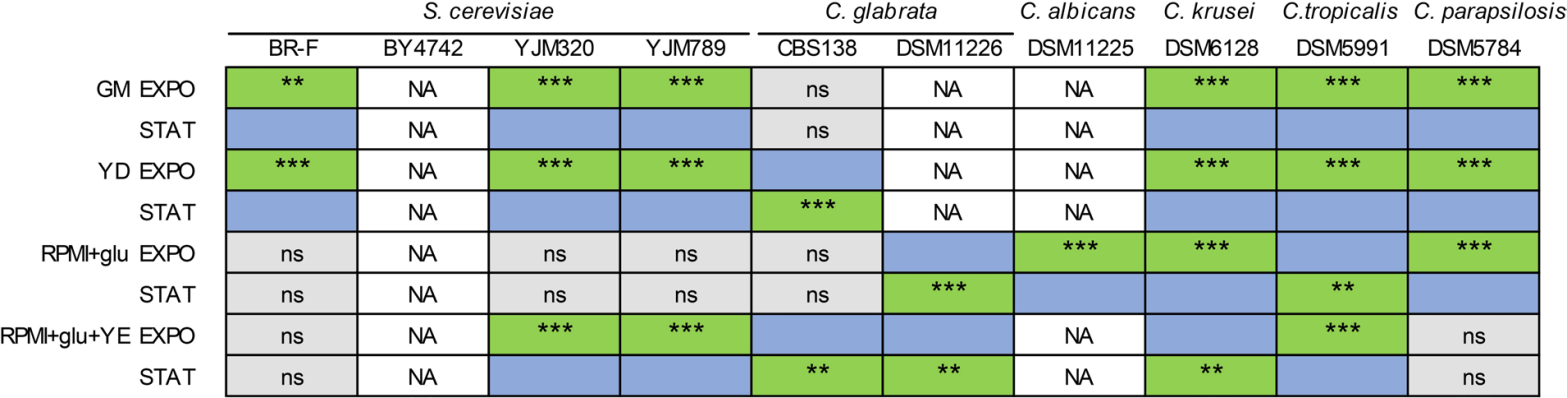
Summary of adhesion differences between exponential- and stationary-phase cells. Overview of adhesion comparisons for individual strains in each medium based on data from Fig. 4. For each exponential–stationary-phase pair, green indicates higher adhesion and blue indicates lower adhesion. Statistical significance is denoted by asterisks (***, *p* < 0.001; **, *p* < 0.01). Gray boxes and “ns” indicate no significant difference in adhesion between exponential-phase and stationary-phase cells. NA indicates cases where adhesion was not detected or remained at background levels.

### Differences in adhesion efficiency between exponential- and stationary-phase cells vary among yeast species and depend on the growth medium

We next compared the adhesion efficiency of exponential- and stationary-phase cells of each strain pre-grown in the individual media (Figs. 4 and 5). Adhesion was quantified using a modified crystal violet staining assay followed by colorimetric measurement of the dye. Absorbance was measured at 592 nm, and adhesion values were expressed as percentages relative to the adhesion of exponential-phase cells of the *S. cerevisiae* strain BR-F grown in the GM medium, which was set to 100%. As expected, neither exponential-phase nor stationary-phase cells of the laboratory strain BY4742 (non-adhesive control strain) adhered to the polystyrene surface of wells in microtiter plates in any of the four media tested. In contrast, cells of all other strains exhibited adhesion when pre-grown in at least one of the tested media (Figs. 4 and 5).

Significant differences in adhesion efficiency between exponential- and stationary-phase cells were observed for several strains; these differences were strain-specific and occurred only under certain growth conditions (Fig. 5). Exponential-phase cells were significantly more adhesive than stationary-phase cells in adhesive *S. cerevisiae* strains grown in the respiratory GM and fermentative YD media (BR-F, YJM320, and YJM789 strains), as well as in RPMI+glu+YE for strains YJM320 and YJM789. In contrast, no significant differences in adhesion between exponential- and stationary-phase cells were detected for these strains when grown in RPMI+glu, or for strain BR-F grown in RPMI+glu+YE (Fig. 4).

A similar pattern – higher adhesion efficiency of exponential-phase cells compared to stationary-phase cells – was observed for *C. krusei*, *C. tropicalis*, and *C. parapsilosis* pre-grown in GM and YD, as well as for *C. parapsilosis* and *C. albicans* pre-grown in RPMI+glu (Fig. 4). In contrast, stationary-phase cells exhibited higher adhesion efficiency than exponential-phase cells in *C. glabrata* CBS when pre-grown in YD and RPMI+glu+YE, and in *C. glabrata* DSM when pre-grown in RPMI+glu and RPMI+glu+YE (Fig. 4). No differences in adhesion between exponential- and stationary-phase cells were observed for *C. glabrata* CBS pre-grown in GM and RPMI+glu, and *C. glabrata* DSM was non-adhesive when pre-grown in GM and YD (Figs. 4 and 5).

**Fig. 6:**
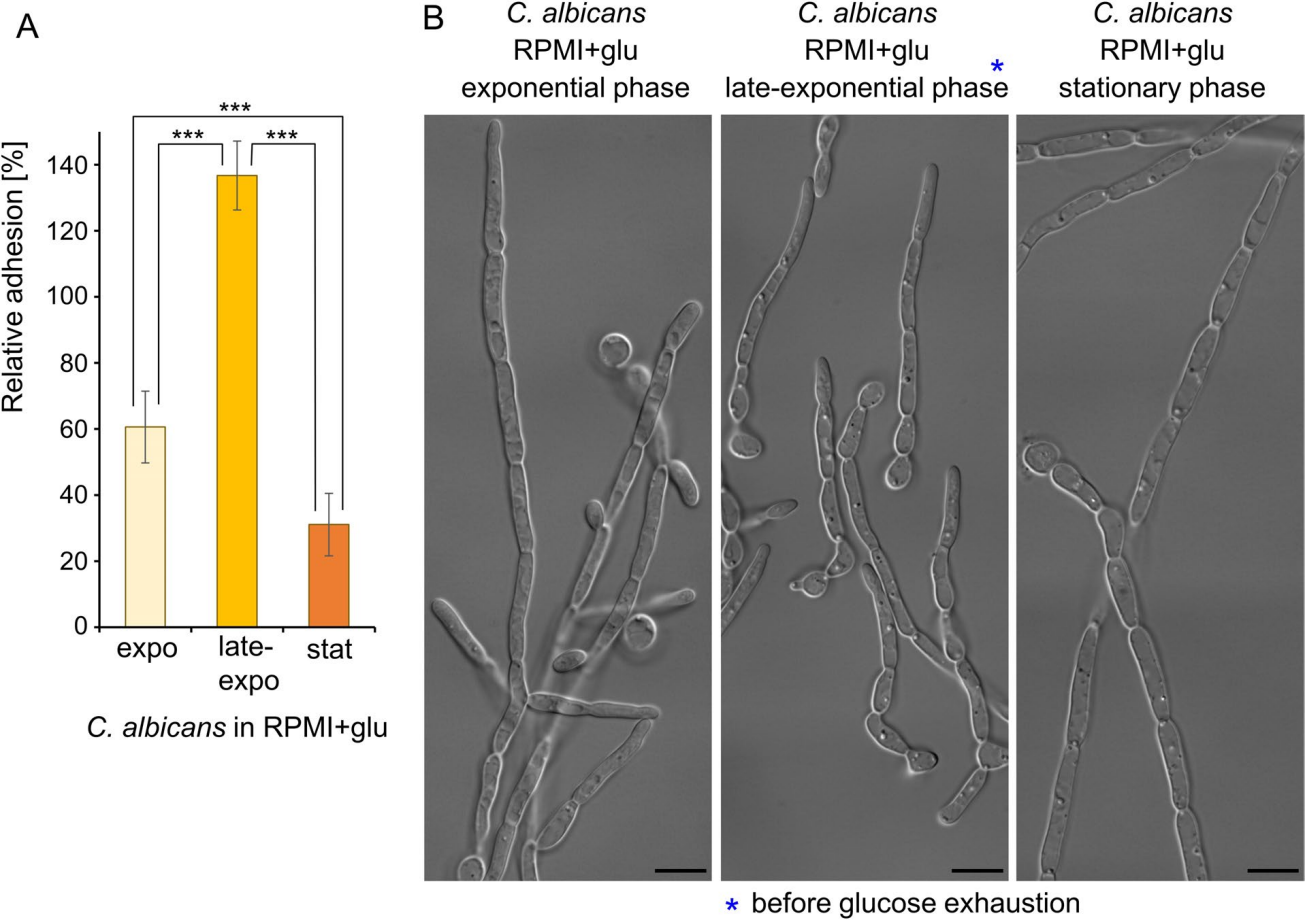
Adhesion of *C. albicans* cells in three growth phases. (**A**) Relative adhesion of cells shown in panel B. Data represent mean values from eight independent replicates with standard deviations (SD). Statistical significance was determined using one-way ANOVA followed by Tukey’s post hoc test for multiple pairwise comparisons: ***, *p* < 0.001. (**B**) Exponential-, late-exponential-, and stationary-phase cells of *C. albicans* grown in RPMI+glu medium visualized by DIC microscopy. Scale bar, 10 μm.

In the case of *C. albicans*, the adhesion efficiency of exponential-phase cells in RPMI+glu reached only approximately 60% of the reference adhesion level of *S. cerevisiae* BR-F exponential cells grown in GM (Fig. 4), and adhesion of stationary-phase cells was even lower (approximately 31%). These values were markedly lower than those previously reported for cells of the same *C. albicans* strain harvested prior to the diauxic shift (i.e., just before glucose depletion), which reached approximately 125% of the adhesion level of *S. cerevisiae* BR-F grown in GM^11^. We therefore directly compared the adhesion of exponential-phase and stationary-phase *C. albicans* cells with that of pre-diauxic cells pre-grown in RPMI+glu (Fig. 6A). Consistent with previous findings, the highest adhesion efficiency was observed in pre-diauxic cells (approximately 136% of the BR-F reference value). Notably, *C. albicans* formed hyphae in all three growth phases analyzed (exponential, pre-diauxic, and stationary) (Fig. 6B).

Overall, adhesion efficiency did not consistently correlate with cell morphology. Both yeast-form cells and pseudohyphae of certain strains exhibited high adhesion efficiency in specific media, as illustrated by strong adhesion of yeast-form cells of *C. glabrata* CBS across all media and by high adhesion of pseudohyphae formed by *C. krusei*. Conversely, both yeast-form cells and pseudohyphae could also display low adhesion efficiency, as observed for *C. glabrata* DSM pre-grown in GM and YD and for stationary-phase cells of *C. parapsilosis* pre-grown in YD and RPMI+glu. A notable exception was *C. albicans*, for which adhesion was detected only in hyphal cells pre-grown in RPMI+glu – the only medium in which *C. albicans* exhibited substantial adhesion. In contrast, yeast-form *C. albicans* cells grown in RPMI+glu+YE, GM, and YD exhibited little or no adhesion.

## Discussion

The ability to adhere to solid or semi-solid surfaces is crucial for many biological activities of yeasts, including the formation of surface-associated, spatially organized biofilms as well as less structured surface coatings on a wide range of natural and artificial materials encountered in environmental, industrial, and biomedical settings^2,3^. Yeast adhesion may, but does not necessarily, be associated with a dimorphic transition from yeast-form to filamentous (pseudohyphal or hyphal) growth and with subsequent invasion of a semi-solid substrate, such as host tissues^23,24^.

Despite the importance of identifying parameters that determine the ability of different yeasts to adhere to solid abiotic surfaces, comparative studies systematically examining multiple physiological factors that influence adhesion to abiotic materials (e.g., plastic, stainless steel, glass, or wood) remain scarce, representing a significant gap in the field^1,25^. Moreover, most studies addressing the role of cell physiology in adhesion to either biotic or abiotic surfaces have focused primarily only on *C. albicans*, the major yeast pathogen, often using diverse cultivation conditions and different biological or artificial substrates^1^. To address these gaps, in this comparative study, we analyzed cell morphology and adhesion to an abiotic polystyrene surface in three adhesive strains of *S. cerevisiae* and six strains representing different *Candida* species, examined in two distinct growth phases and four media.

A clear correlation between adhesion and a specific cellular morphology was observed only in *C. albicans*. When pre-grown in GM, YD, or RPMI+glu+YE, *C. albicans* DSM11225 formed predominantly yeast-shaped cells, which either did not adhere to the polystyrene surface above the level of the negative control *S. cerevisiae* BY4742 (GM and YD) or showed very low adhesion (RPMI+glu+YE). In contrast, growth in nutrient-poor RPMI+glu medium induced the formation of hyphae that adhered to polystyrene with relatively high efficiency. This finding is consistent with earlier studies reporting enhanced adhesion of germinating *C. albicans* cells or elongated hyphae to biotic surfaces, such as epithelial cells. For example, germinated *C. albicans* cells grown in Sabouraud glucose broth or in tissue culture medium M199 adhered more efficiently to epithelial or buccal cells than non-germinated cells^26^. Similarly, adhesion of *C. albicans* grown in Williams medium to epithelial cells increased with the elongation of germ tubes and hyphae^27^. The hypha-specific surface adhesin Als3, which mediates adhesion and whose encoding gene has no close orthologs in some other *Candida* spp.^28^, may be involved in the observed specificity of hyphal adhesion in *C. albicans*. On the other hand, when *C. albicans* adhesion was tested under flow instead of static conditions, all three morphological forms – hyphal, pseudohyphal, and yeast-like – were able to adhere to the endothelium, with the yeast-like form adhering in the highest numbers^29^. The physical conditions under which adhesion occurs are therefore another important factor influencing the adhesion of different cell forms.

In contrast to *C. albicans* DSM11225, no consistent correlation between cell adhesion efficiency and cellular morphology was detected for the other yeast species analyzed in this study. Both yeast-form cells and pseudohyphae or hyphae of certain strains (pre-grown in specific media) were able to adhere to the polystyrene surface. Our data thus challenge the widespread assumption that filamentation universally enhances adhesion in yeasts and show that cell morphology alone cannot serve as a general predictor of adhesion capacity across yeast species.

Comparison of adhesion to polystyrene across the four media confirmed that adhesion efficiency varies substantially among strains and growth conditions^11,30^ and revealed pronounced differences between exponential- and stationary-phase cells. The direction and magnitude of these differences were species-specific and further modulated by the medium (Fig. 5). Where significant differences were observed, most yeast species – including all *S. cerevisiae* strains, *C. albicans*, and *C. parapsilosis* – showed higher adhesion efficiency of exponential-phase cells compared to stationary-phase cells. In addition, in *C. albicans*, cells harvested prior to diauxic shift (i.e., late exponential-phase cells before glucose depletion) adhered even more efficiently than mid-exponential cells. A similar trend was reported for adhesion of late-exponential *C. albicans* cells grown in yeast nitrogen base (YNB) with glucose to HeLa cells, or in Sabouraud–glucose medium to Vero cells^31^. However, when alternative carbon sources (YNB with maltose, sucrose, mannose, or galactose, or glucose–peptone medium) were used, no clear relationship between *C. albicans* growth phase and adhesion was observed^31^.

A different trend was observed for both *C. glabrata* strains: where significant differences were observed, stationary-phase cells always adhered more efficiently than exponential-phase cells. This observation is consistent with the function of Epa6 and Epa7 adhesins (GPI-anchored lectins in the cell wall)^32^, with Epa6p mediating strong hydrophobic interactions that contribute to *C. glabrata* adhesion to abiotic surfaces^33^. *EPA6* and *EPA7* genes are located in subtelomeric regions and repressed by subtelomeric silencing, which must be relieved for their expression^34^. *EPA6* and *EPA7* are expressed in stationary-phase cells and during biofilm formation^32^.

In *C. tropicalis* and *C. krusei*, the relationship between growth phase and adhesion efficiency was medium-dependent. Exponential-phase cells of *C. tropicalis* pre-grown in GM, YD, and RPMI+glu+YE, as well as *C. krusei* cells pre-grown in GM, YD, and RPMI+glu, adhered more efficiently than stationary-phase cells. However, in the remaining medium for each species (*C. tropicalis* in RPMI+glu and *C. krusei* in RPMI+glu+YE), stationary-phase cells exhibited higher adhesion efficiency than exponential-phase cells.

Only a limited number of studies have directly compared adhesion between exponential- and stationary-phase yeast cells. Some earlier reports showed that exponential-phase *C. albicans* cells adhere less efficiently to epithelial cells than stationary-phase cells, a trend opposite to what we observed for adhesion of *C. albicans* to abiotic polystyrene. For example, stationary-phase *C. albicans* blastospores pre-grown in 1% Phytone peptone broth (a papaic digest of soybean meal, pH 6.9) supplemented with 0.1% glucose adhered more strongly to epithelial cells than logarithmic-phase cells^35^. Higher adhesion of stationary-phase *C. albicans* cells compared to early exponential-phase cells to epithelial surfaces was reported for cells grown in Sabouraud dextrose broth^36^. Lontsi Djimeli reported higher adhesion of stationary-phase *C. albicans* cells to polyethylene under static conditions, whereas under dynamic (stirring) conditions, adhesion was comparable between growth phases^37^. Also adhesion of the brewing strain *S. cerevisiae* to the mica surface was higher in cells from the stationary growth phase compared to cells from the logarithmic phase (grown in MYGP - malt medium)^38^.

The opposite trends observed for adhesion to biotic materials (e.g., epithelial cells) and to different abiotic plastics (polyethylene versus polystyrene used in this study) likely reflect differences in surface physicochemical properties combined with growth phase-dependent changes in yeast cell wall composition. Indeed, early stationary-phase *C. albicans* cells were shown to adhere differently to various materials, including glass, polyethylene terephthalate, polymethyl methacrylate, polystyrene, and polytetrafluoroethylene^39^, although exponential-phase cells were not analysed in that study.

Together, these findings highlight that the relationship among cell physiology, growth phase, and adhesion is highly context-dependent and shaped by both surface properties and environmental conditions. Growth phase-dependent factors, such as remodeling of the cell wall architecture, differential adhesin expression, and differences in lipid metabolism that affect membrane properties, may contribute to variations in cell surface physicochemical properties and cell adhesion to abiotic surfaces. Further studies are needed to identify factors (genetic and biophysical) that distinguish exponential- and stationary-phase cells in individual species and explain the contrasting adhesion patterns observed, such as those between *C. glabrata* and other yeast species.

## Materials and methods

### Yeast strains and growth media

The yeast strains used in this study are listed in Table 1. Liquid media included the respiratory GM medium (3% glycerol, 1% yeast extract), and glucose-based media YD (2% glucose, 1% yeast extract), RPMI+glu (RPMI 1640 with glutamine and phenol red, with bicarbonate [LM-R1640, BIOSERA, France]) supplemented with D-glucose to a final concentration of 2%, pH adjusted to 5.6 with 1 M HCl^40^, RPMI+glu+supplements (RPMI+glu with 50 mg/l of histidine, leucine, and lysine, and 20 mg/l of uracil) for cultivation of *S. cerevisiae* BY4742 auxotroph, and RPMI+glu+YE (RPMI+glu supplemented with 1% yeast extract).

### Cell cultivation

#### Growth characteristics of yeast strains

 Cells of individual yeast strains from glycerol stocks (stored at − 75 °C) were grown on YPDA (2% glucose, 1% yeast extract, 1% peptone and 2% agar) plates for 24 h and then inoculated into liquid media to an initial density of OD_600_ = 0.005–0.08 (measured using a Helios Gamma spectrophotometer, Unicam), depending on strain growth rate and medium composition, and cultivated with shaking at 150 rpm overnight at 28 °C in an Infors HT Ecotron shaking incubator for approximately 14 h. Cells from these precultures were then transferred to 50 ml pre-warmed fresh medium of the same composition in 250 ml Erlenmeyer flasks to an initial density of OD_600_ = 0.1. Cultures were incubated at 28 °C with shaking and OD_600_ was monitored over a 30 h period to obtain growth curves. Generation time was calculated from the exponential growth phase of each curve. Fold increase in biomass was expressed as the relative increase in OD_600_, calculated as the ratio of OD_600_ at 25 h of cultivation (t_25_; stationary phase for all strains) to OD_600_ at time 0 (t_0_).

#### Preparation of cells for adhesion assays

For adhesion experiments, cells from YPDA plates were inoculated into liquid media (OD_600_ = 0.005–0.08), depending on strain growth rate and medium composition, and cultivated overnight at 28 °C for approximately 14 h. Cells from these precultures were transferred to fresh medium of the same composition to an initial density of OD_600_ = 0.1 and cultivated at 28 °C either to the exponential growth phase (5 h in YD and RPMI+glu+YE, or 6 h in GM and RPMI+glu) or to the stationary phase (27 h in all media). At these time points (see also Fig. 1), cell samples were collected for adhesion assays and microscopy.

### Adhesion assays and data processing

Exponential- and stationary-phase cells were harvested as described above, washed with sterile distilled water, and resuspended in sterile water to an OD_600_ = 1. Aliquots of 150 µl of the cell suspension were transferred to wells of 96-well polystyrene microtiter plates, with four independent replicates per condition. Plates were incubated at 28 °C with shaking at 150 rpm for 3 h to allow cell adhesion.

Adhesion to polystyrene was quantified using a modified crystal violet–based assay^11^ adapted from previously published methods^21,41,42^. After incubation, non-adherent cells were removed and wells were washed three times with distilled water. Each well was then incubated with 150 µl of 1% crystal violet solution at room temperature with gentle shaking for 15 min. Excess dye was removed, wells were washed three times with distilled water, and plates were air-dried for 30 min. Crystal violet retained by adherent cells was eluted with 150 µl of a 2:1 (v/v) mixture of 96% ethanol and distilled water for 1 min. Absorbance at 592 nm (A_592_) was measured using an Epoch microplate spectrophotometer (BioTek, Agilent, USA) after transferring 100 µl of the eluted solution to a new microtiter plate. Absorbance values reflected the amount of adherent cells and were used as a measure of adhesion efficiency. Background staining was determined in control wells processed without cells and subtracted from all measured values.

Relative adhesion was calculated relative to the adhesion of the *S. cerevisiae* BR-F strain pre-grown in GM medium, which was set to 100%. Standard deviations were calculated from at least eight replicates. Statistical significance of differences in adhesion between exponential- and stationary-phase cells within a given medium was assessed using a two-tailed unpaired t-test (Fig. 4). Differences in adhesion among the three growth stages of *C. albicans* in RPMI+glu were evaluated using one-way ANOVA followed by Tukey’s post hoc test for multiple pairwise comparisons (Fig. 6). Statistical analyses were performed using GraphPad Prism 6. P values ≤ 0.05 were considered statistically significant (*, *p* < 0.05; **, *p* < 0.01; ***, *p* < 0.001).

### Cell staining and microscopy

#### Cell staining

 Lipid droplets were stained using Nile Red, a lipophilic dye used for lipid droplets visualization^17,43^. A Nile Red stock solution (0.2 µg/ml in DMSO) was diluted tenfold, mixed 1:1 with cell samples, and incubated 15 min in the dark before microscopy.

#### Microscopy

For morphological analysis, differential interference contrast (DIC) images were acquired using a Leica DMR microscope equipped with HCX PL Fluotar 100x/1.30 Oil objective, filter sets for DIC and Jenoptik ProgRes MF Cool camera, using NIS-Elements (AR 4.51.01 64-bit) software. Fluorescence imaging was performed using a Carl Zeiss Axio Observer.Z1 inverted fluorescence microscope equipped with Plan-Apochromat 63×/1.40 Oil DIC M27 objective and Axiocam 506 camera, using ZEN 2012 (blue edition) software. Filter set for red fluorescence (Nile Red; excitation 538–562 nm; emission 570–640 nm), DIC, or bright-field imaging were used.

## Data Availability

This study does not generate data deposited in external repositories. The datasets used and/or analysed during the current study are available from the corresponding author on reasonable request.
